# Japanese multicenter registry evaluating the antegrade dissection reentry with cardiac computerized tomography for chronic coronary total occlusion

**DOI:** 10.1007/s12928-021-00762-x

**Published:** 2021-02-07

**Authors:** Maoto Habara, Etsuo Tsuchikane, Kazuki Shimizu, Yoshifumi Kashima, Kenichiro Shimoji, Shigeru Nakamura, Takeshi Niizeki, Takaki Tsutsumi, Yoshiaki Ito, Tomohiro Kawasaki, for the Bridge point Club Japan

**Affiliations:** 1 Department of Cardiovascular Medicine, Toyohashi Heart Center, 21-1 Gobudori, Oyama-cho, Toyohashi, Aichi 441-8530 Japan; 2 Department of Radiology, Toyohashi Heart Center, Toyohashi, Aichi Japan; 3 Department of Cardiovascular Medicine, Sapporo CardioVascular Clinic, Sapporo, Hokkaido Japan; 4 Department of Cardiology, Saiseikai Utsunomiya Hospital, Utsunomiya, Tochigi Japan; 5 Department of Cardiology, Kyoto Katsura Hospital, Kyoto, Japan; 6 Department of Cardiology, Okitama Public General Hospital, Yamagata, Japan; 7 Department of Cardiology, Saga Medical Center Kouseikan, Saga, Japan; 8 Department of Cardiology, Saiseikai Yokohama-City Eastern Hospital, Yokohama, Kanagawa Japan; 9 Department of Cardiology, Cardiovascular Center Shin-Koga Hospital, Kurume, Fukuoka Japan

**Keywords:** Antegrade dissection reentry, Chronic total occlusion, Cardiac computed tomography angiography

## Abstract

Recently, antegrade dissection re-entry (ADR) with re-entry device for chronic total occlusion (CTO) percutaneous coronary intervention (PCI) has evolved to become one of the pillar techniques of the hybrid algorithm. Although the success rate of the device is high, it could be improved. We sought to evaluate the current trends and issues associated with ADR in Japan and evaluate the potential of cardiac computed tomography angiography (CCTA) for ADR procedure. A total 48 patients with CTO suitable for ADR evaluated by baseline conventional angiography and CCTA were enrolled. Procedural success and technical success were evaluated as the primary and secondary observations. Furthermore, all puncture points were analyzed by CCTA. CT score at each punctured site depended on the location of plaque deposition (none; + 0, at isolated myocardial site; + 1, at epicardial site; + 2) and the presence of calcification (none; + 0, presence; + 1) was analyzed and calculated (score 0–3). Overall procedure success rate was 95.8%. Thirty-two cases were attempted with the ADR procedure and 25 cases of them were successful. The technical success rate was 78.1% and myocardial infarction or other major complications were not observed in any cases. CT score at 60 puncture sites in 32 cases were analyzed and the score at technical success points was significantly smaller compared to that at technical failure points (0.68 ± 1.09 vs 1.77 ± 1.09, *p* < 0.0001). CTO-PCI with Stingray device in Japan could achieve a high procedure success and technical success rate. Pre procedure cardiac CT evaluation might support ADR procedure for appropriate patient selection or puncture site selection.

## Introduction

Percutaneous coronary intervention (PCI) of chronic total occlusions (CTO) is a rapidly evolving field. The initial success rate is increasing with the improvements in technology and technique, such as the retrograde approach [1]. Recently, antegrade dissection and re-entry (ADR) for CTO-PCI has also evolved to become one of the techniques, especially in the US and parts of Europe [2, 3]. Although there are several ADR techniques, including subintimal tracking and re-entry (STAR) or the limited antegrade subintimal tracking (LAST) technique, recent ADR can be achieved with the CrossBoss Catheter (Boston Scientific, Marlborough, Massachusetts) and the Stingray System (Boston Scientific) [4]. Especially in hybrid operations, they have become an essential device and are used in 20–34% of cases in their registries [4–7]. And now, ADR with the Stingray device is one of the cornerstones of the hybrid algorithm [2, 3]. Unlike previous ADR techniques, such as the STAR or LAST technique, ADR with the Stingray System is not associated with a marked increase in major adverse cardiac events (MACE) [7] and the success rate could be improved to 86% [4]. However, the success rate remains not always high especially as first crossing strategy [5, 8, 9] and Stingray use has remained low outside the US [5,10–15. This means patient or lesion selection might be important to raise the technical success rate of ADR. Previous reports showed that factors associated with high risk for failure included smaller vessel size, severe calcification, severe tortuosity, and side-branch involvement [4]. Furthermore, other manuscripts analyzed intravascular ultrasound (IVUS) and showed thick intimal thickness at the puncture site contributes to the failure of re-entry [16]. Cases with a diffuse narrowing distal true lumen with plaque may not be good candidates for ADR and the target position for ADR should be selected at a healthy looking distal true lumen without plaque.

Coronary CT angiography (CCTA) is a recently useful less-invasive tool that demonstrates high performance for diagnosis and prognosis of coronary artery disease [17–19]. Especially in CTO case, pre-procedural CCTA is a useful imaging modality to defining the most optimal treatment strategies and to raise the success rate [20–22]. And we thought that it could detect vessel size, calcification, tortuosity, side-branch involvement, and possibly thick intimal thickness. Therefore, pre-procedural CCTA images may help us to select patients, lesion and suitable re-entry site for the ADR procedure with Stingray and might contribute to raise the success rate of ADR. Although CrossBoss and Stingray had not been available in Japan, they became available in 2017. To examine the safety and effectiveness of ADR procedure in Japan, we established a scientific organization called the BridgePoint Club Japan. This study aims to analyze current trends and issues associated with ADR in Japan and evaluate the potential of CCTA imaging for patient selection or puncture site selection of ADR.

## Methods

### Study population

We constructed a multicenter, prospective, nonrandomized registry of patients attending 30 Japanese centers between April 2017 and April 2019. Patients with total occlusion (Thrombolysis In Myocardial Infarction [TIMI] flow grade 0) of major epicardial coronary vessel clinically determined to be > 3 months old were considered for this study. Patients had to report angina or have ischemia documented on a stress test. All patients detected a de novo CTO lesion by angiography were also performed CCTA.

Patient exclusion criteria included left ventricular ejection fraction < 20%, serum creatinine > 2 mg/dl without hemodialysis, vein graft or in-stent CTO lesion, allergy to aspirin or all thienopyridines, aorto-ostial lesion. In addition, angiographic including CCTA exclusion criteria were patients with adverse coronary angiographic features for ADR as severe tortuosity, lack of a visible distal vessel true lumen, CTO involving a large side branch (angiographic landing zone < 10 mm proximal to any major bifurcation). Lesion length was not considered in study eligibility. Although no patients in this series were turned down for an attempt at recanalization because of adverse angiographic features, the patient selection highly depended on the availability of this device. The protocol was approved by ethics committees in each participating center, and all participants gave written informed consent.

### Coronary CT angiography

All patients received CCTA before PCI procedure in each hospital. Unless contraindicated, an intravenous or oral dose of metoprolol was given to target a heart rate of < 65 beats/min, and sublingual nitroglycerin was administered before CCTA. These CCTA data were sent to Toyohashi Heart Center and were analyzed by single highly experienced reader (K.S). The assessment was performed blinded to all clinical data. The CT data sets were transferred to an offline workstation (Aquarious NetStation, Terarecon Inc., San Mateo, California) for image analysis. The optimal display image setting was adjusted for each patient at a window from 600 to 900 HU and a level from 40 to 250 HU. Before the PCI, severe calcification or severe tortuosity of proximal to CTO lesion and side-branch involvement was evaluated for appropriate patient selection for ADR, similar to angiographic inclusion criteria.

### PCI procedure

After successful arterial access with one or two sheaths was accomplished, a heparin bolus was given to achieve an activated clotting time (ACT) > 300 s. Bilateral coronary injections to visualize collateral circulation were utilized unless antegrade collaterals provided excellent details of the distal lumen. A 6-8Fr guiding catheter for antegrade manner was selected at the operator’s discretion. All cases were started by the antegrade approach with single wire manipulation. The wire and micro-catheter included CrossBoss Catheter (Boston Scientific, Marlborough, Massachusetts) selection, or wire escalation and step down were operator’s decision. When the wire could not cross to the distal vessel true lumen, ADR procedure with the Stingray Balloon was attempted. Therefore, parallel wire technique was not attempted in all cases. The puncture position was decided by angiography with contralateral injection at the time. To advance the Stingray Balloon to the required position, antegrade rotational microcatheter (Corsair Asahi Intecc, Nagoya, Aichi, Japan) has dilated the passage as the bougie technique. If the microcatheter could not pass, small balloon (1.0–1.5 mm) dilatation was performed and then the microcatheter was advanced again. After the microcatheter reached the required position, the guide wire was changed to a supportive wire, such as Miracle 12 g (Asahi Intecc, Aichi, Japan). And the microcatheter was changed to the Stingray Balloon and advanced to optimal position to perform ADR. Contralateral injection and coronary angiography in appropriate angles were utilized to confirm the optimal position of Stingray Balloon and to determine the orientation of Stingray Balloon exit ports above or below, right or left of the true lumen.

###  < Method 1; April 2017-June 2018 > 

**Fig. 1:**
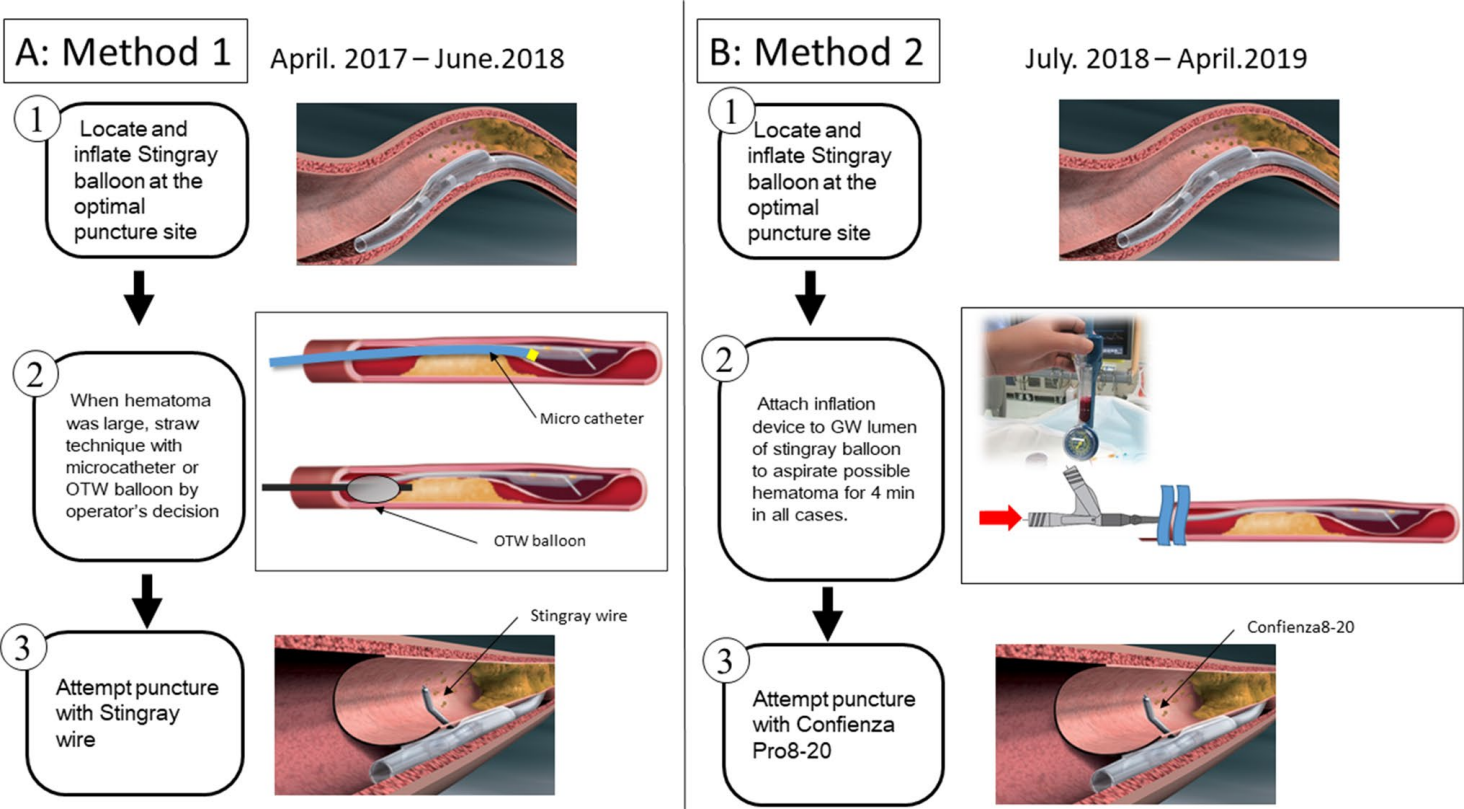
Detail of antegrade dissection re-entry (ADR) procedure. **a** Method1: applied from April 2017 to June 2018. After located and inflated stingray balloon at the optimal puncture site, punctured by stingray wire. However, when hematoma was large, straw technique with microatheter or over-the-wire (OTW) balloon was performed by operator’s decision. **b** Method2: applied from July 2018 to April 2019. After located and inflated stingray balloon at the optimal puncture site, aspirate hematoma attached by inflation device for 4 min used in all cases regardless of hematoma size. After the aspiration, puncture with confienza pro 8–20^®^ (Asahi intec., Japan) not stingray wire was attempted

Stick and swap technique (Stick means punctured by stiff wire from subintimal space to make the pathway toward the true lumen and the wire is then removed and change the polymer-jacketed wire. Swap means the polymer-jacketed wire is subsequently advanced through the same exit port and the pathway) [23] was attempted in all cases with the Stingray wire for stick, XT-R (Asahi Intecc, Aichi, Japan) for swap. When the ADR puncture was difficult because of large hematoma, the straw technique with a small microcatheter or over-the-wire balloon was attempted [24]. (Fig. 1a).

###  < Method 2; July 2018-April 2019 > 

After the Stingray Balloon reached the target position, an inflation device was attached to the guide wire lumen of Stingray Catheter to aspirate possible hematoma for 4 min in all cases whether hematoma was detected or not. Subsequently, stick and swap technique was attempted in all cases with Confienza 8–20 (Asahi intecc, Aichi, Japan) for stick, XT-R for swap. (Fig. 1b).

After ADR puncture succeeded, IVUS was performed to confirm the puncture point. When the ADR procedure failed, retrograde approach or IVUS guided re-wiring were attempted by the operator’s decision.

Patients were hospitalized overnight and discharged the next day if there was no MACE. MACE included all death, emergent bypass surgery, repeated revascularization, Q wave or non-Q wave myocardial infarction (MI), any vascular complications, and contrast-induced nephropathy (CIN). Documentation of new, pathological Q waves in 2 or more contiguous leads in an electrocardiogram associated with any elevation of creatine kinase-MB was required for a diagnosis of Q wave myocardial infarction. Non-Q wave myocardial infarction was defined as the elevation of creatine kinase to more than twice the upper limit associated with any elevation of creatine kinase-MB without the appearance of Q waves. CIN was defined as either > 25% increase of serum creatinine or an absolute increase in serum creatinine of 0.5 mg/dL from baseline value at 24 h following the exposure to contrast media. Baseline patient characteristics, procedural details and techniques, and in-hospital outcomes were recorded. The primary observation was procedural success, which was defined as both guidewire and balloon crossing a completely occluded lesion, successfully dilation of the occluded artery, and restoration of antegrade flow (TIMI flow grade 3) with < 50% residual stenosis on final angiography. Secondary observations included technical success, which was defined as successful revascularization using this device system (successful puncture using Stingray Balloon Catheter).

### IVUS, CCTA analysis

**Fig. 2:**
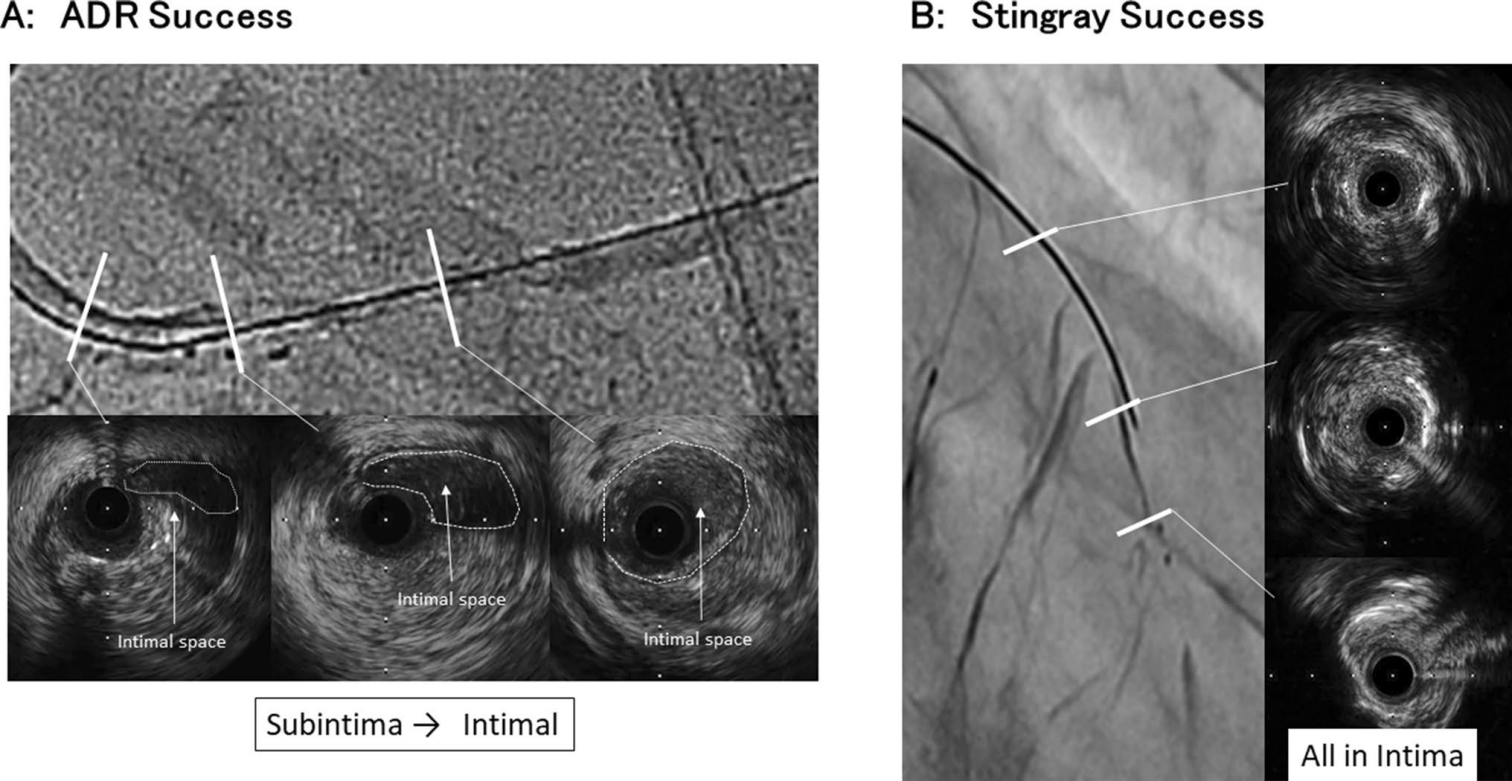
Intravascular ultrasound (IVUS) image cases with subintimal based success and intimal based success. **a** Case with chronic total occlusion (CTO) in right coronary artery succeeded by antegrade dissection and re-entry (ADR) procedure. IVUS images showed that guide wire crossed from proximal subintimal space to distal intimal pace. White dot circles indicate intimal space as true lumen. **b** Case with CTO in left anterior descending artery succeeded by ADR procedure. IVUS images showed guide wire crossed thorough intimal space even though ADR procedure with Stingray device was performed after antegrade wire escalation failure

All IVUS studies were performed after successful ADR procedure. All IVUS images were reviewed and evaluated by an independent, experienced analyst (Sho Yamashita) who was blinded to clinical and angiographic lesion characteristics to confirm puncture point. According to the IVUS image, they were classified into two groups as (1): Subintimal-based success group and (2): Intimal-based success success group. Subintimal-based success was defined as IVUS showed that guide wire cross from proximal subintimal space at the CTO lesion into distal vessel true lumen (Fig. 2a). On the other hand, Intimal-based success was defined as IVUS showed the wire was located all intra plaque at CTO lesion (Fig. 2b). In subintimal-based success group, thickness of intima at re-entry site was measured.

**Fig. 3:**
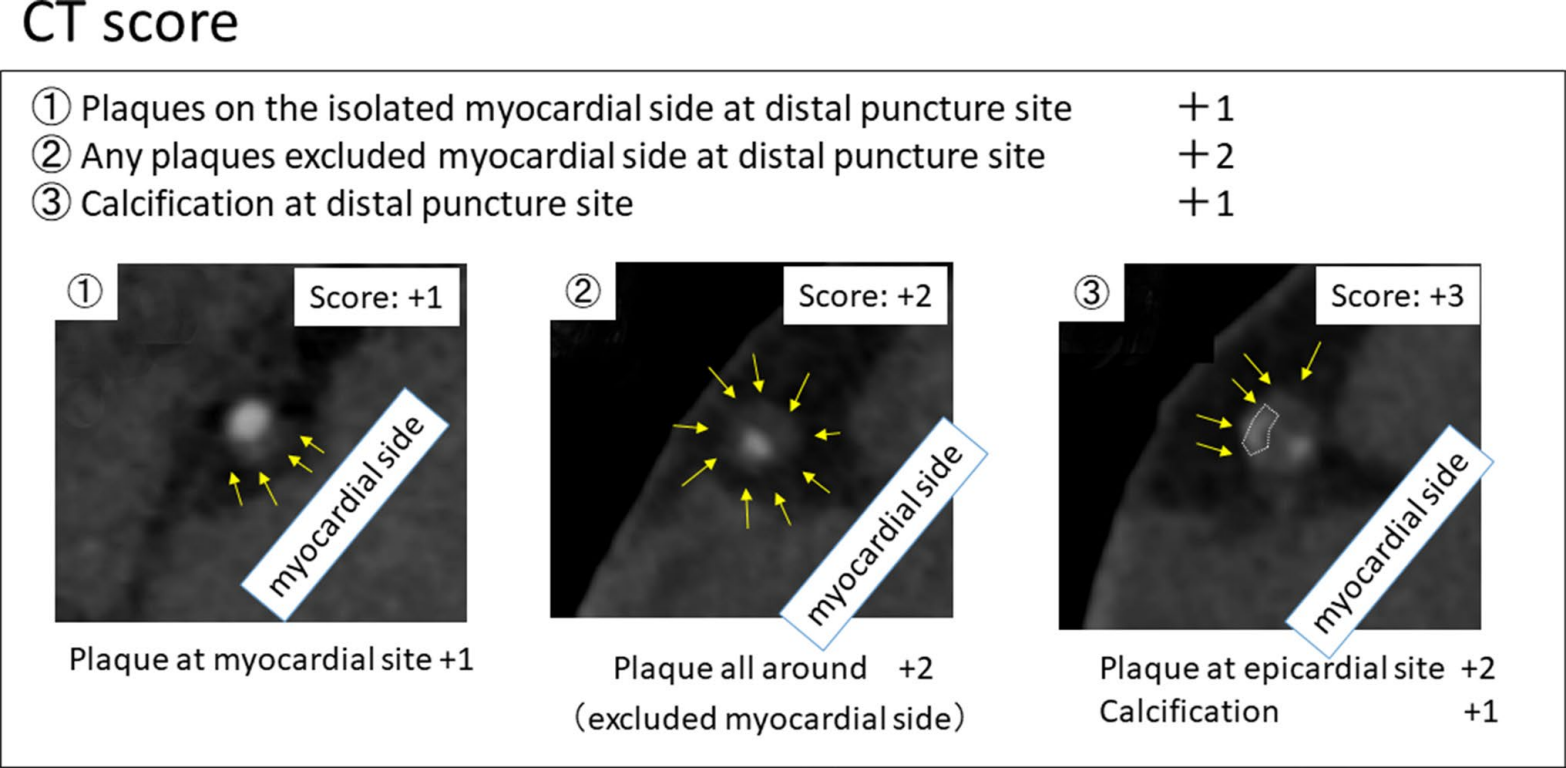
Definition of computed tomography (CT) score at puncture site. ①–③: Cross sectional images of coronary CT angiography at antegrade dissection re-entry (ADR) puncture sites. CT score was calculated based on the definition. White arrows indicated the plaque. White dot lines shows the calcification

**Fig. 4:**
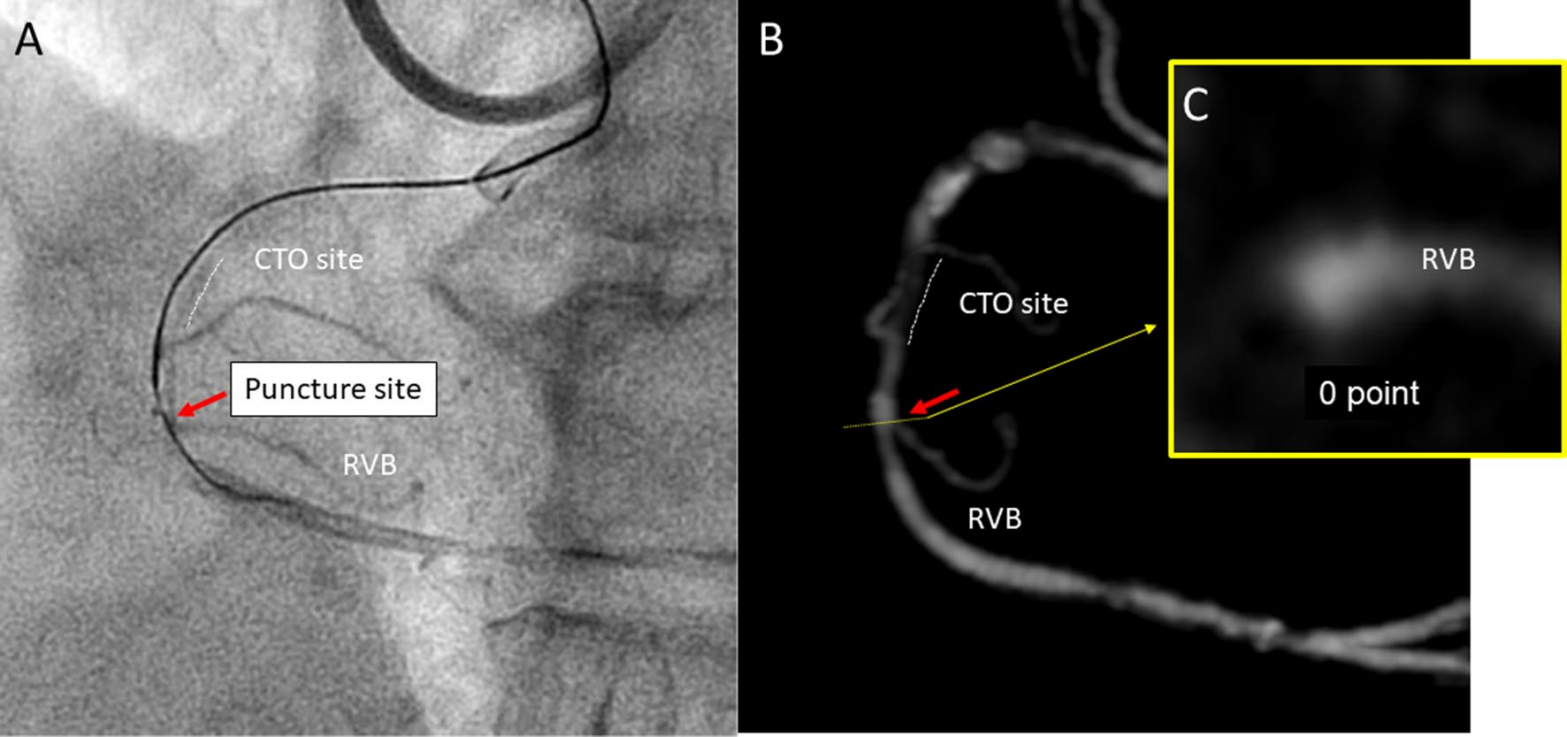
Example of computed tomography (CT) score evaluation at puncture site. Case with chronic total occlusion (CTO) in right coronary artery succeeded by antegrade dissection re-entry (ADR) procedure. **a** Angiography when stick with confienza8-20 and stingray balloon was performed. Red arrow indicates the puncture point. **b** Curved image of coronary CT. Red arrow indicate puncture point beside the right ventricular branch selected according to the percutaneous coronary intervention angiographic image. **c** CT score was calculated using by cross-sectional image at the site

To investigate the influence of CCTA on ADR procedure, we evaluated the presence of plaque or calcification at puncture site on distal true lumen. In each point, (1) plaques on the isolated myocardial side at distal puncture site (+ 1 point), (2) any plaques beyond the myocardial side (+ 2 points), (3) Calcification on both 1 and 2 at distal puncture site (+ 1 point) was analyzed and calculated as the CT score (Score 0–3) (Fig. 3). It was analyzed by an independent expert (Kazuki Shimizu) after the PCI. All cases which attempted ADR were analyzed about puncture sites in distal vessel true lumen. All puncture sites both successful and failed were analyzed based on cross-sectional images and curved multiplanner reconstruction images. According to the curved image, puncture points were selected according to the PCI angiographic images and scoring was performed using by cross-sectional images. Detail and examples were showed in Fig. 4. Side branches, ostia and significant calcification were used as landmarks to ensure corresponding puncture site on CCTA as on PCI angiographic images.

### Statistical analysis

Continuous variables are expressed as the means ± standard deviation. Categorical data are expressed as the number or frequency of occurrence. Comparison of continuous variables was performed by the nonparametric Mann–Whitney U test. The chi-squared test or Fisher’s exact test for sparse data was used for comparison of the frequency of occurrence. The SPSS version 11.0 software (SPSS, Inc., Chicago, Illinois) was used for data analysis. A *p* value of < 0.05 was considered statistically significant.

## Results

### Patient and lesion characteristics

**Fig. 5:**
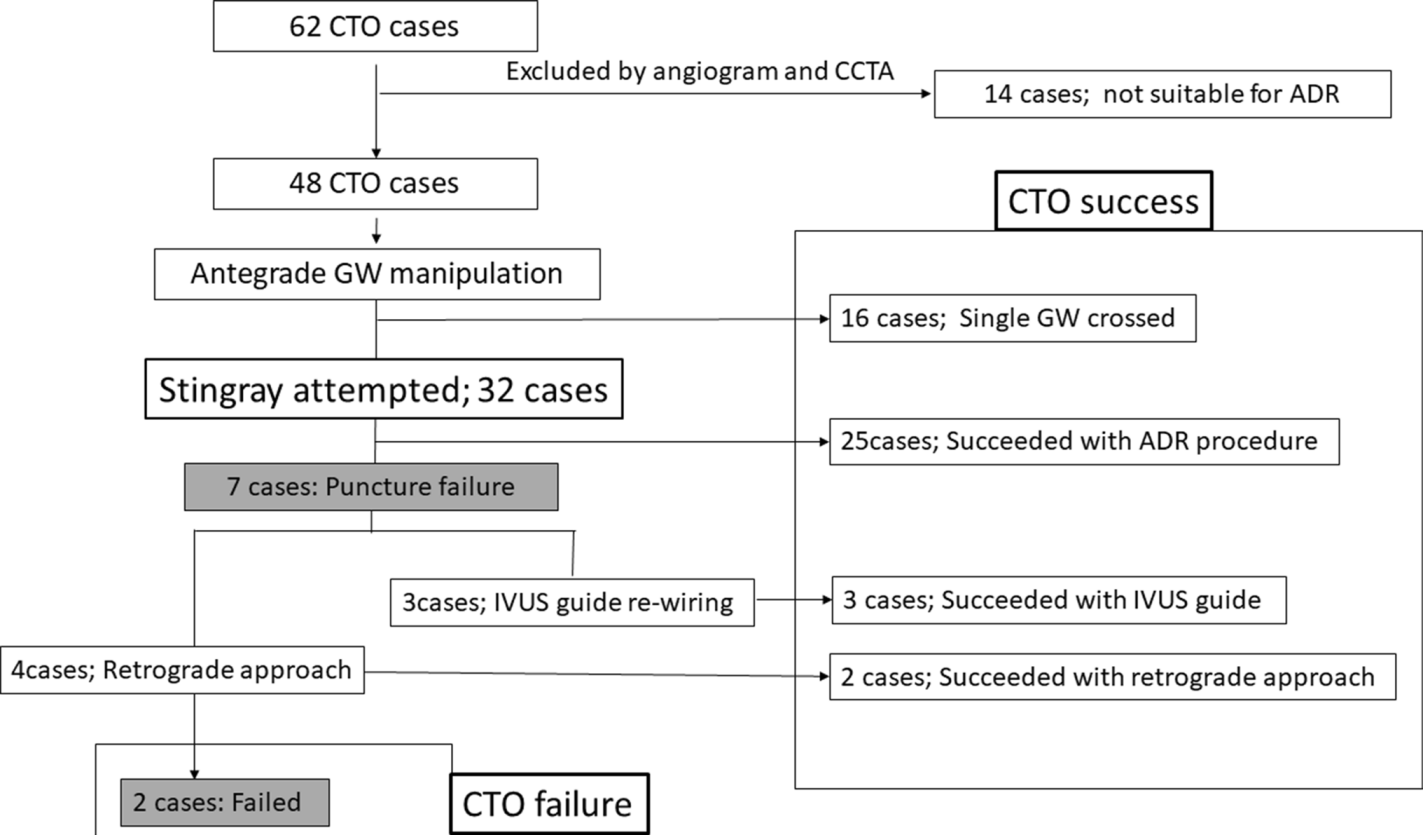
Patient flow chart. *CCTA* cardiac computed tomography angiography, *CTO* chronic total occlusion, *GW* guide wire, *ADR* antegrade dissection and re-entry, *IVUS* Intravascular ultrasound

Sixty-two patients with total occlusion suitable for revascularization were evaluated by baseline coronary angiography and considered to enroll in this study from April 2017 to April 2019 from 30 enrolled centers. However, after careful evaluation of CCTA and coronary angiography by expert operator (E.T), 14 cases were excluded (3 cases: include major side branch, 3 cases: sub-total lesion and not CTO, 4 cases: distal vessel true lumen for re-entry was too distal of vessel, 3 cases: primary retrograde approach should be recommended because there are remarkable interventional collateral, 1 case: low cardiac function) and finally 48 cases treated by 27 operators were enrolled to this study (Fig. 5). Patient demographics and lesion characteristics were shown in Table 1.

**Table 1:** Patient and lesion characteristics

Patient demographics	*n* = 32
Male	29 (90.6%)
Age	67.3 ± 10.4
Prior MI	8 (25%)
Prior CABG	2 (6.3%)
Prior PCI	19(59.4%)
HF	8(25.0%)
Hemodialysis	0 (0%)
HT	28 (87.5%)
HL	25 (78.1%)
DM	11 (34.4%)
(Insulin)	1 (3.1%)
Smoking	17 (53.1%)
(Current)	7 (21.9%)
Family history	5 (15.6%)
LVEF	60.2 ± 11.7
Pre procedure Cre	0.9 ± 0.2
Pre procedure eGFR	69.4 ± 20.3

Lesion characteristics	*n* = 32
RCA/LAD/LCX	16/14/2
Blunt	6 (18.8%)
Calc	20 (62.5%)
Tortuosity > 45	9 (28.1%)
CTO length > 20 mm	10 (32.3%)
Re-try	8 (25.0%)
J-CTO score	1.7 ± 1.0

*MI* myocardial infarction, *CABG* coronary artery bypass graft, *PCI* percutaneous coronary intervention, *HT* Hypertension, *HL* Hyperlipidemia, *DM* diabetes mellitus, *EF* ejection fraction, *eGFR* estimated glomerular filtration rate, *RCA* right coronary artery, *LAD* left anterior descending artery, *LCX* left circumflex artery, *CTO* Chronic total occlusion

### Primary and secondary observations

Within 48 CTO cases, 16 cases were successful with antegrade single guide wire manipulation. Therefore, 32 cases (66.7%) were attempted ADR with stingray balloon. 25 cases were puncture successful and seven cases failed. After the puncture failure, three cases were successful with IVUS guide re-wiring and two cases were successful with retrograde approach. Finally, two cases were in-procedure failure. Overall procedure success rate was 95.8% (46/48) and antegrade success rate was 91.3% (42/46). Technical success rate with Stingray was 78.1% (25/32). The mean procedure time was 169.3 ± 83.9 min, the radiation dose and contrast volume were 4.0 ± 2.7 Gy and 206.5 ± 92.1 ml.

Comparison analysis between patients with technical success (*n* = 25) and technical failure (*n* = 7) is shown in Table 2. Although there was no significant difference between the two groups regarding patient characteristics, there was a remarkable difference in lesion characteristics. And there was also a different tendency (*p* = 0.05) about the procedure characteristic between mothod1 and method2.

**Table 2:** Comparison analysis between patient with technical success and technical failure

	Technical success *n* = 25	Technical failure *n* = 7	*p*
Patient characteristics			
Male	22 (88%)	7 (100%)	0.46
Age	67.8 ± 9.6	65.7 ± 13.6	0.65
Prior MI	5 (20%)	3 (42.9%)	0.22
Prior CABG	2 (8%)	0 (0%)	0.61
Prior PCI	15 (60%)	4 (57.1%)	0.61
HF	7 (28%)	1 (14.3%)	0.42
Hemodialysis	0 (0%)	0 (0%)	–
HT	22 (88%)	6 (85.7%)	0.65
HL	19 (76%)	6 (85.7%)	0.51
DM	7 (28%)	4 (57.1%)	0.16
(Insulin)	1 (4%)	0 (0%)	0.78
Smoking	12 (48%)	4 (57.1%)	0.25
(Current)	4 (16%)	3 (42.9%)	0.16
Family history	5 (20%)	0 (0%)	0.26
LVEF	60.2 ± 11.7	57.7 ± 13.4	0.64
Pre procedure Cre	0.88 ± 0.23	0.84 ± 0.23	0.72
Pre procedure eGFR	67.1 ± 17.5	77.4 ± 28.3	0.24
Lesion characteristics			
*RCA*	12 (48%)	4 (57.1%)	0.88
*LAD*	11 (44%)	3 (42.9%)	
*LCX*	2 (8%)	0 (0%)	
Blunt	4 (16%)	5 (71.4%)	0.39
Calc	16 (64%)	4 (57.1%)	0.54
Tortuosity > 45	4 (16%)	5 (71.4%)	**0.01**
CTO length > 20 mm	7(28%)	3 (42.9%)	0.38
Re-try	6 (24%)	2 (28.6%)	0.58
J-CTO score	1.5 ± 1.0	2.3 ± 0.8	**0.04**
Procedure characteristics			
Mehod 1	11 (44%)	6 (85.7%)	**0.05**
Method 2	14 (56%)	1 (14.3%)	
Puncture times	1.6 ± 0.8	2.9 ± 0.8	** < 0.001**
MACCE	0 (0%)	1(14%)	0.21
Coronary perforaiton	0 (0%)	1(14%)	0.21
Tamponade	0 (0%)	0 (0%)	–
Non ST elevation MI	0 (0%)	0 (0%)	–
ST elevation MI	0 (0%)	0 (0%)	–
Stroke	0 (0%)	0 (0%)	–-
CIN	0 (0%)	0 (0%)	–
Death	0 (0%)	0 (0%)	–

*MI* myocardial infarction, *CABG* coronary artery bypass graft, *PCI* percutaneous coronary intervention, *HT* Hypertension, *HL* Hyperlipidemia, *DM* diabetes mellitus, *EF* ejection fraction, *eGFR* estimated glomerular filtration rate, *RCA* right coronary artery, *LAD* left anterior descending artery, *LCX* left circumflex artery, *CTO* Chronic total occlusion, *MACE* Major adverse cardiac event, *CIN* contrast-induced nephropathy

### Complications and in-hospital adverse events

**Fig. 6:**
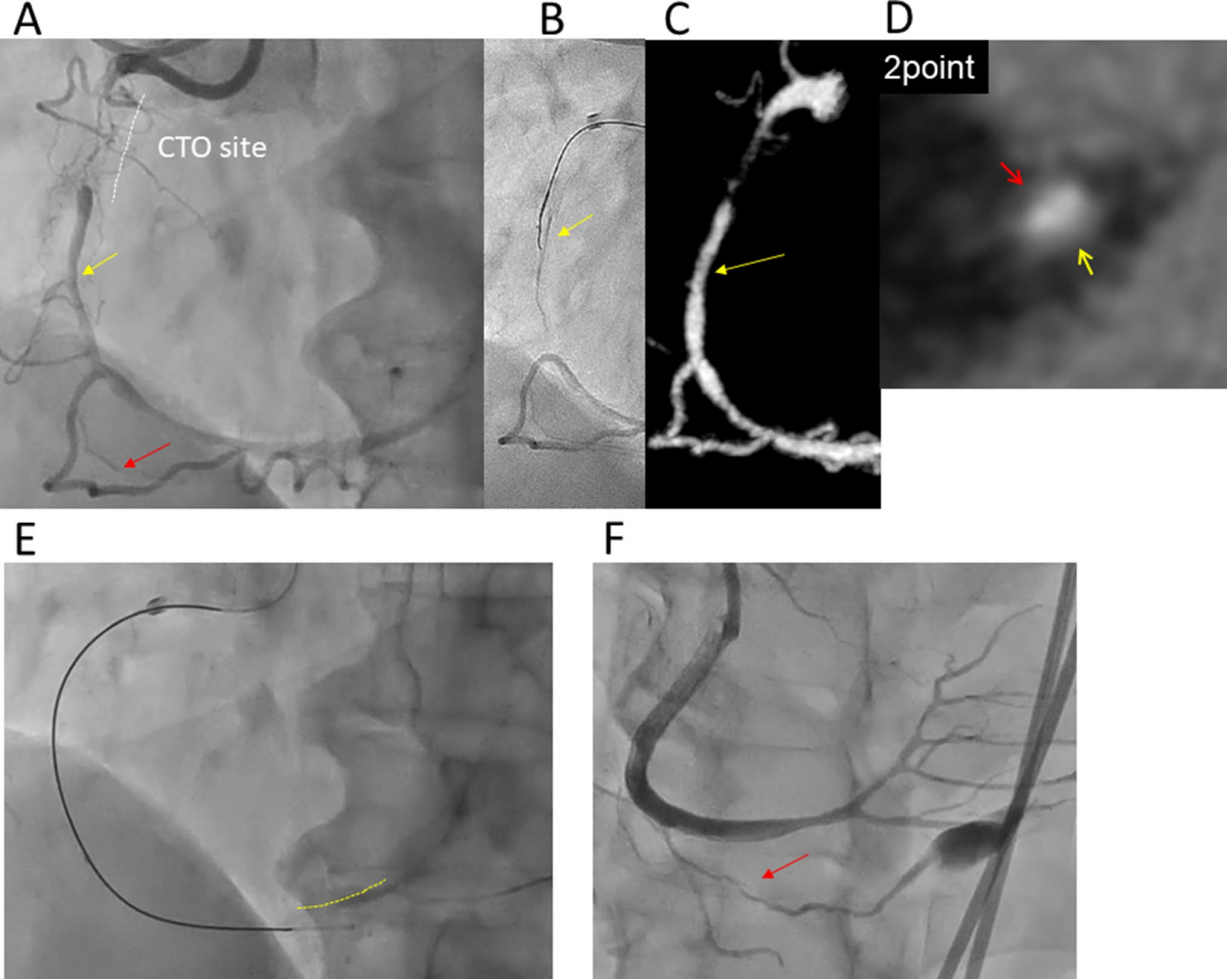
Case with coronary perforation with CrossBoss catheter. **a** Angiography showed chronic total occlusion (CTO) lesion at proximal right coronary artery (RCA). **b** Puncture with stingray wire and stingray balloon was performed at mid RCA (Yellow arrow), but it was failed. **c** Curved image of coronary computed tomography (CT). Yellow arrow indicated the 1^st^ puncture point according to the PCI angiographic image. **d** Cross sectional image at the puncture site and CT score was 2 point. Red arrow indicates the plaque at epicardial site and yellow arrow indicated the plaque at myocardial site. **e** After failure of the 1st puncture, CrossBoss catheter advanced to distal. However, the device was assumed to cross through outside of vessel because distal true lumen (Yellow dot line) visualized by contralateral angiography was completely far from the device. **f** Retrograde procedure was performed to bail out. The angiography after stent implantation showed coronary perforation because of CrossBoss cateter penetration into small branch of right ventricular branch (Red arrow). The case was treated without cardiac tamponade due to coil insertion into the branch

Complications during the procedure were observed in only one case. In that case, coronary perforation without cardiac tamponade caused by the CrossBoss Catheter was observed (Fig. 6). Fortunately, successful bailout was accomplished using the retrograde approach without any other complications. The case was the first case in this registry. Therefore, no other PCI cases were performed using the CrossBoss Catheter. No MACE including myocardial infarction was observed in all cases. There was no significant difference about complications and adverse events between patients with Stingray technical success and failure (Table 2).

### Cardiac CT and IVUS analysis

Cardiac CT analyzed 60 puncture sites in 32 cases which were attempted ADR with Stingray System (1.88 sites/case) and shown in Table 3. No plaque image was more frequently observed and CT score was significantly smaller at puncture succeeded points compare to puncture failure points.

**Table 3:** Comparison analysis of cardiac computed tomography angiography at distal puncture site

Plaque distribution	Puncture success *n* = 25	Puncture failure *n* = 35	*p*
None	15 (60.0%)	5 (14.3%)	**0.0002**
At myocardial site	6 (24.0%)	7 (20.0%)	0.76
All around	4 (16.0%)	23 (65.7%)	**0.0003**
With calcification	3 (12.0%)	9 (25.7%)	0.19
**CT score**	0.68 ± 1.09	1.77 ± 1.09	** < 0.0001**

	Puncture success *n* = 25		
Plaque distribution	Intimal-based success *n* = 9	Subintimal-based success *n* = 16	*p*
None	5 (55.6%)	10 (62.5%)	0.73
At myocardial site	2 (22.2%)	4 (25.0%)	0.88
All around	2 (22.2%)	2 (12.5%)	0.52
With calcification	2 (22.2%)	1 (6.3%)	0.24
**CT score**	0.89 ± 1.09	0.75 ± 1.06	0.4

*CT* computed tomography, *ADR* antegrade dissection re-entry

IVUS analysis was performed only 25 cases which were guide wire successfully crossed with Stingray system. Intimal-based success was observed in nine cases (36%) and subintimal-based success was observed 16 cases (64%). Although the IVUS images were significantly different, CT score and CT plaque distribution were similar between intimal-based success and subintimal-based success group (Table 3). Mean intimal thickness at puncture sites in patients with subintimal-based success (*n* = 16) was 0.3 ± 0.2 mm.

## Discussion

The main findings of this registry were (1) CTO-PCI with ADR procedure in Japan could achieve a high procedure and technical success rate, (2) CCTA might be useful not only for patient selection but also for optimal puncture site selection evaluated by plaque distribution and presence of calcification at distal true lumen, (3) improvement of puncture wire and hematoma management with the Stingray Balloon and inflation device might contribute to the technical success rate, and (4) from IVUS analysis, not all cases performed ADR procedure with the Stingray System were observed guide wire crossing from subintimal space to intimal space after careful antegrade wiring.

To adapt the ADR device in Japanese, there were some problems about particular complications to be solved, such as coronary perforation or myocardial infarction. In FAST-CTOs, coronary perforation occurred in 9.3% and myocardial infarction occurred in 4.3% [4]. Those were a slightly higher compared to our previous registry [11, 12]. Most of reasons of the perforation in ADR procedure would be associated with CrossBoss Catheter. Actually, in our first case, massive perforation because of CrossBoss advancement into small side branch was observed (Fig. 6). It would appear that the complication with CrossBoss cannot be avoided, because it is usually impossible to control the device in fine-pitch movement not only short axis direction, but also long axis direction. Therefore, we do not recommend the use of CrossBoss device during the CTO procedure except in-stent occlusion. Our style is based on antegrade guide wire manipulation. Therefore, when that fails, the wire has already reached a subintimal position parallel to the distal cap and the CrossBoss catheter is usually not needed. In fact, the Stingray Balloon could be reached to optimal position without the CrossBoss device in all cases attempted ADR procedure except the first case, which observed coronary perforation with the CrossBoss. Bougie technique with rotational microcatheter like Corsair and small balloon dilatation would be enough to dilate the passage for Stingray Balloon [25].

ADR procedure with Stingray system also carries the risk of periprocedural myocardial infarction because of the occlusion of coronary side branches or collateral circulation. Therefore, patient and lesion selection must be important to avoid this complication. CTO lesion involving the major side branch should be not suitable for the ADR procedure. In the search for innovative imaging modalities with improved visualization of CTO when compared with conventional coronary angiography, CCTA has emerged as an accurate less-invasive tool to assess the trajectory, morphology and CTO length as well location the vessel distal to the occlusion which often may not be well seen on conventional angiography [26]. Therefore, both coronary angiography and CCTA should be used for patient and lesion selection for ADR procedure. In our registry, all cases were performed coronary angiography and CCTA before PCI procedure and only suitable cases for ADR procedure were selected to enroll. This patient/lesion selection with angiography and CCTA might have contributed to our result of no cases with MACE including myocardial infarction. In addition, our data showed vessel tortuosity is also one of the features for technical failure of ADR. Thus, CTO lesion with tortuosity also should not be selected for this procedure.

Another limitation of ADR procedure is that success rate is not high as first attempted strategy (Progress CTO: 52.5%, Recahrge registry: 67%)[5, 9]. This means the puncture with Stingray device is not always easy. Our data showed, even in technical success group, number of punctures were 1.6 ± 0.8 times. One of the difficulties of the puncture was assumed to be due to the difficulty to detect the optimal reentry zone as plaque less site by conventional angiography only. Although multiple factors seem to be affected the suitability of a reentry zone (calcification, plaque load, lumen size, plaque thickness, distance of first wire to true lumen, hematoma size, distal lumen pressure, tortuosity of zone stability of the Stingray Balloon, and operator experience) [25], previous IVUS data showed the thick intimal thickness at puncture site contributes to the failure of re-entry [16]. Therefore, figuring out where is plaque less and non-calcified zone at distal true lumen before intervention has importance to simplify the puncture procedure [27]. During the last decade, CCTA has emerged as a valuable less-invasive tool to evaluate not only coronary stenosis, calcification or vessel trajectory but also non-obstructive coronary plaque or plaque characterization [27–29]. Therefore, we made the CT score based on the existence of plaque and calcification at distal true lumen and the number of CT score could was associated with the technical success rate. According to the result, the pre-procedure evaluation of CT score at distal true lumen might help ADR procedure. Furthermore, puncture method including changing the puncture guide wire and kinds of management hematoma may associate with technical success rate. Especially puncture guide wire with stronger penetration force/efficacy might be important because Stingray puncture guide wire sometimes prolapses and fails when we stick.

In IVUS analysis showed guide wires were crossed through intra plaque after ADR procedure in 36% of cases. This means, not all cases adapted Stingray System after careful antegrade wiring were performed subintiamal stenting.

### Limitations

This study had several limitations. First, the major limitation of this study was the selection bias. The enrolled cases were very selected by angiography and CCTA. In addition, J-CTO score was smaller compared to previous registries [5, 9, 12]. Therefore, the outcomes of this study may not be achieved in a real-world clinical practice for all CTO lesions. Second, in this registry, parallel wire technique after single guide wire manipulation was skipped because parallel wiring sometimes increases the risk of hematoma formation and makes subsequent Stingray very difficult [25]. Thus, selection criteria to commence Stingray or parallel wire technique after antegrade escalation failure cannot be described. Finally, lack of mid- and long-term follow-up, beyond the duration of hospital stay, is also an important limitation of this study.

## Conclusion

CTO-PCI with Stingray device in Japan could achieve a high procedure success rate (95.8%) and high technical success rate (78.1%). Pre-procedure Cardiac CT and CT score evaluation might support ADR procedure for appropriate patient selection or puncture site selection.

## References

[1] Rathore S, Matsuo H, Terashima M (2009). Procedural and inhospital outcomes after percutaneous coronary intervention for chronic total occlusions of coronary arteries 2002 to 2008: Impact of novel guidewire techniques. JACC Cardiovasc Interv.

[2] Michael TT, Papayannis AC, Banerjee S (2012). Subintimal dissection/reentry strategies in coronary chronic total occlusion interventions. Circ Cardiovasc Interv.

[3] Brilakis ES, Grantham JA, Rinfret S (2012). A percutaneous treatment algorithm for crossing coronary chronic total occlusions. JACC Cardiovasc Interv.

[4] Whitlow PL, Burke MN, Lombardi WL (2012). Use of a novel crossing and re-entry system in coronary chronic totalo cclusions that have failed standard crossing techniques: results of the FAST-CTOs (facilitated antegrade steering technique in chronic total occlusions) trial. JACC Cardiovasc Interv.

[5] Maeremans J, Dens J, Spratt JC (2017). Antegrade dissection and reentry as part of the hybrid chronic total occlusion revascularization strategy: a subanalysis of the RECHARGE registry (registry of crossboss and hybrid procedures in France, the Netherlands, Belgium and United Kingdom). Circ Cardiovasc Interv.

[6] Christopoulos G, Karmpaliotis D, Alaswad K (2015). Application and outcomes of a hybrid approach to chronic total occlusion percutaneous coronary intervention in a contemporary multicenter US registry. Int J Cardiol.

[7] Azzalini L, Dautov R, Brilakis ES (2017). Procedural and longer-term outcomes of wire- versus device-based antegrade dissection and re-entry techniques for the percutaneous revascularization of coronary chronic total occlusions. Int J Cardiol.

[8] Danek BA, Karatasakis A, Karmpaliotis D (2016). Use of antegrade dissection re-entry in coronary chronic total occlusion percutaneous coronary intervention in a contemporary multicenter registry. Int J Cardiol.

[9] Tajti P, Karmpaliotis D, Alaswad K (2018). The hybrid approach to chronic total occlusion percutaneous coronary intervention: update from the PROGRESS CTO registry. JACC Cardiovasc Interv.

[10] Brilakis ES, Mashayekhi K, Tsuchikane E (2019). Guiding principles for chronic total occlusion percutaneous coronary intervention. Circulation.

[11] Habara M, Tsuchikane E, Muramatsu T (2016). Comparison of percutaneous coronary intervention for chronic total occlusion outcome according to operator experience from the Japanese retrograde summit registry. Catheter Cardiovasc Interv.

[12] Suzuki Y, Tsuchikane E, Katoh O (2017). Outcomes of percutaneous coronary interventions for chronic total occlusion performed by highly experienced japanese specialists: the first report from the Japanese CTO-PCI expert regist. JACC Cardiovasc Interv.

[13] Wu EB, Tsuchikane E, Ge L (2020). Retrograde versus antegrade approach for coronary chronic total occlusion in an algorithm-driven contemporary asia-pacific multicenter registry: comparison of outcomes. Heart Lung Circ.

[14] Sapontis J, Salisbury AC, Yeh RW (2017). Early procedural and health status outcomes after chronic total occlusion angioplasty: a report from the OPEN-CTO registry (outcomes, patient health status, and efficiency in chronic total occlusion hybrid procedures). JACC Cardiovasc Interv.

[15] Konstantinidis NV, Werner GS, Deftereos S (2018). Temporal trends in chronic total occlusion interventions in Europe. Circ Cardiovasc Interv.

[16] Tsuchikane E, Kimura M, Suzuki T (2012). New re-entry device for revascularization of chronic coronary total occlusions: preliminary single Japanese center experience. J Invasive Cardiol.

[17] Budoff MJ, Dowe D, Jollis JG (2008). Diagnostic performance of 64-multidetector row coronary computed tomographic angiography for evaluation of coronary artery stenosis in individuals without known coronary artery disease: results from the prospective multicenter ACCURACY (Assessment by Coronary Computed Tomographic Angiography of Individuals Undergoing Invasive Coronary Angiography) trial. J Am Coll Cardiol.

[18] Miller JM, Rochitte CE, Dewey M (2008). Diagnostic performance of coronary angiography by 64-row CT. N Engl J Med.

[19] Meijboom WB, Meijs MF, Schuijf JD (2008). Diagnostic accuracy of 64-slice computed tomography coronary angiography: a prospective, multicenter, multivendor study. J Am Coll Cardiol.

[20] Opolski MP, Achenbach S, Schuhbäck A (2015). Coronary computed tomographic prediction rule for time-efficient guidewire crossing through chronic total occlusion: insights from the CT-RECTOR multicenter registry (Computed Tomography Registry of Chronic Total Occlusion Revascularization). J Am Coll Cardiol Intv.

[21] Opolski MP, Achenbach S (2015). CT angiography for revascularization of CTO crossing the borders of diagnosis and treatment. JACC Cardiovasc Imaging.

[22] Opolski MP, Hartaigh ÓB, Berman DS (2015). Current trends in patients with chronic total occlusions undergoing coronary CT angiography. Heart.

[23] Christopoulos G, Kotsia AP, Brilakis ES (2015). The double-blind stick-and-swap technique for true lumen reentry after subintimal crossing of coronary chronic total occlusions. J Invasive Cardiol.

[24] Smith EJ, Di Mario C, Spratt JC (2015). Subintimal TRAnscatheter Withdrawal (STRAW) of hematomas compressing the distal true lumen: a novel technique to facilitate distal reentry during recanalization of chronic total occlusion (CTO). J Invasive Cardiol.

[25] Wu EB, Brilakis ES, Lo S (2020). Advances in CrossBoss/Stingray use in antegrade dissection reentry from the Asia Pacific Chronic Total Occlusion Club. Catheter Cardiovasc Interv.

[26] Hoe J (2009). CT coronary angiography of chronic total occlusions of the coronary arteries: how to recognize and evaluate and usefulness for planning percutaneous coronary interventions. Int J Cardiovasc Imaging.

[27] Ghoshhajra BB, Takx RAP, Stone LL (2017). Real-time fusion of coronary CT angiography with X-ray fluoroscopy during chronic total occlusion PCI. Eur Radiol.

[28] Motoyama S, Ito H, Sarai M (2015). Plaque characterization by coronary computed tomography angiography and the likelihood of acute coronary events in mid-term follow-up. J Am Coll Cardiol.

[29] Cho YK, Nam CW, Koo BK (2018). Usefulness of baseline statin therapy in non-obstructive coronary artery disease by coronary computed tomographic angiography: From the CONFIRM (COronary CT Angiography EvaluatioN For Clinical Outcomes: An InteRnational Multicenter) study. PLoS ONE.

